# Particle Tracking Methods for Battery Precipitation Reactions

**DOI:** 10.1021/acs.jpcc.6c00310

**Published:** 2026-05-21

**Authors:** Trent Koberna, Steven C. DeCaluwe

**Affiliations:** 3557 Colorado School of Mines Department of Mechanical Engineering, 1500 Illinois St, Golden, Colorado 80401, United States

## Abstract

Precipitation and deposition reactions at solid–liquid interfaces play a key role in a number of battery chemistries, including Li-ion, so-called “anode free” batteries, zinc-based battery chemistries, and lithium–sulfur, among others. Although models with heterogeneous nucleation and growth phenomena are present in the literature, papers have not to date provided much detail on the numerical algorithms used to track the temporal evolution of the particle size distribution of deposits on electrode surfaces. In this paper we examine several approaches to discretize and track the particle size distribution, demonstrating that common approaches lead to anomalous flattening of the particle size distribution. We conclude by presenting an algorithm that preserves the appropriate particle size distribution during particle growth.

## Graphical abstract

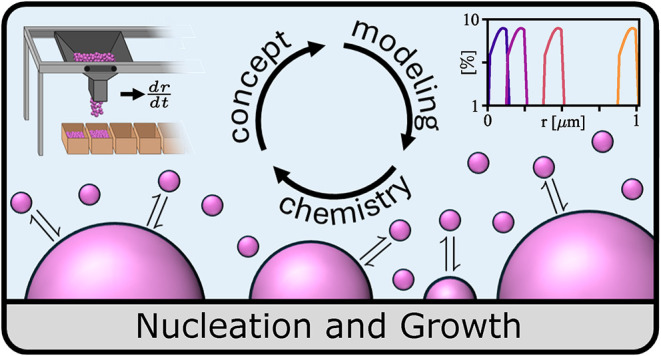

## 1 Introduction

Precipitation and deposition reactions play key roles in a number of “beyond Li-ion” battery applications. Specific examples include:

- *Anode-free Li Metal*: During charging in so-called “anode-free” batteries, Li metal deposits form and grow on a bare copper current collector.^1^ The nucleation and growth dynamics significantly impact the Li morphology, which directly controls device durability.^2–6^

- *Lithium–sulfur*: In lithium–sulfur batteries, polysulfide species dissolved in the liquid electrolyte are reduced during discharge, depositing and growing on the cathode surface as low-order polysulfide (e.g., Li_2_S) precipitates. The capacity and rate capability are sensitive to the precipitate nucleation density.^7–9^

- *Lithium–air*: In lithium–air batteries, oxygen that dissolves into the liquid electrolyte from a cathode flow channel is reduced during discharge, depositing and growing on the cathode surface as lithium oxide (Li_2_O) and peroxide (Li_2_O_2_). The precipitate morphology plays a significant role in determining the overall cathode utilization and porous cathode transport properties during battery cycling, which significantly impact battery performance.^10,11^

Such deposition reactions typically proceed through two steps.^12^ New precipitates first nucleate at the liquid–solid interface. This creates new material interfaces (precipitate-substrate and precipitate-liquid), and therefore includes a significant energy barrier.^13^ As such, nucleation typically requires supersaturation of the liquid solution, with the nucleation rate (and thus the number of deposited nuclei) increasing with the degree of supersaturation.^14^ After nucleation, growth proceeds with a rate that is proportional to the number of nuclei deposited. Because the growth energy barrier is lower than that for nucleation, the degree of supersaturation typically decreases during growth, such that nucleation rates are typically low during the growth phase.^1^

Correctly modeling precipitate deposition and growth is critical to predicting device performance and degradation for efficient and durable devices. Numerous studies have incorporated heterogeneous nucleation and growth (HNG) kinetics into device simulations.^6,9,13,15–21^ However, there is less detail in the literature describing the method by which these models track the particle size distribution (PSD) of surface precipitates in these systems. This paper evaluates some common approaches to PSD tracking and proposes an algorithm to properly track the PSD during HNG, using a simplified kinetic mechanism to clearly evaluate the PSD accuracy.

## 2 Model Framework

### 2.1 Kinetic Model Assumptions

For easy-to-interpret results, we implement a relatively simple kinetic model to track *N*_i_, the number of particles per unit area with average radius *r*_i_. Assumptions include:

- *Nucleation phase*: Hemispherical nuclei with radius *r*_nuc_ = 0.5 nm are deposited at a rate *q̇*_nuc_^″^ from *t* = 0 s to *t* = 0.5 s.
- *Particle growth*: Once a particle is formed, it grows at a constant rate of $\frac{dr}{dt}=0.25\;\mu m\;s^{-1}$ .

The simulation inputs have the same order of magnitude as real-world parameters. This growth rate is equivalent to a deposition rate of *q̇*_growth_^″^ = 2 × 10^–3^ mol m^–2^ s^–1^. As shown in Figure 1, *q̇*_growth_^″^ is per unit area between existing particles and the surrounding liquid solution. Although larger particles have more available surface area for growth (more mol per second deposited), this growth is also spread over that same area. In this study, we assume that *q̇*_growth_^″^ does not vary with particle size, which results in a $\frac{dr}{dt}$ that is also invariant with particle radius (as illustrated in Figure 1). Therefore, as particles grow, the PSD shape should not change, providing an easy metric to evaluate the internal consistency of the PSD tracking algorithm.

**Figure 1:**
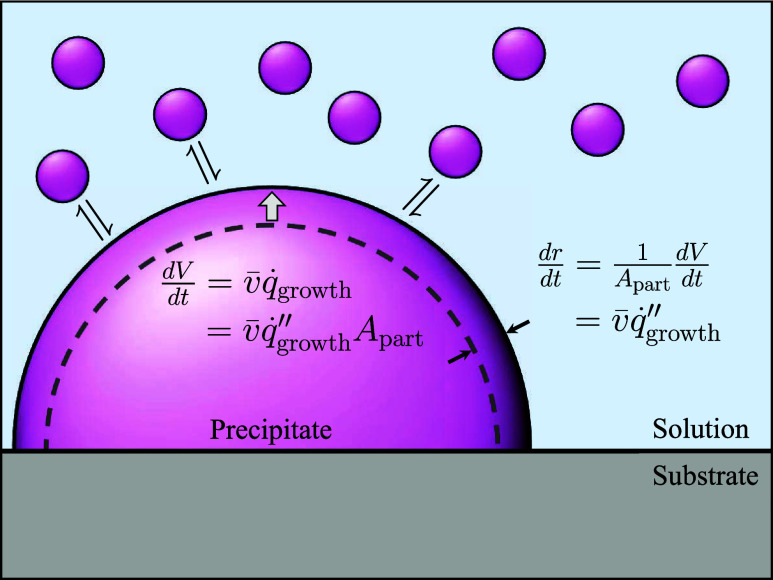
Herein, we assume hemispherical particles and a constant molar deposition rate *q̇*_growth_^″^. This results in a radial growth rate $\frac{dr}{dt}$ that does not depend on particle size, providing a simple means of evaluating the PSD tracking scheme’s accuracy.

## 3 Results and Discussion

The model discretizes the PSD into uniform bins of fixed differential radius Δ*r*. The following discussion focuses on particle growth, but the same principles apply to stripping reactions. Herein, we explore three separate PSD tracking algorithms:

### 3.1 Algorithm 1: Simple Finite Differencing

Our first approach uses finite differencing to track the number of particles entering and exiting each bin. All bins have the same thickness Δ*r*_bin_, and the centroid of each bin represents a fixed radius. Therefore, at any point in time the variable *N*_*i*_ in the solution vector represents the number density of particles with radius between Δ*r* × *i* and Δ*r* × (*i* + 1). The model is defined by a set of ordinary differential equations describing the temporal evolution of *N*_*i*_ for 0 ≤ *i* ≤ *n*_bins_, where *n*_bins_ is the total number of bins. The first bin’s radius equals the nucleation radius: *r*_nuc_.

1 $$r_{0}=r_{\mathrm{nuc}}=\frac{\Delta r_{\mathrm{bin}}}{2}$$

Nucleation only occurs in bin 0. The nucleation rate *q̇*_nuc_^″^, and the fraction of the total surface area *f*_A_ available for deposition (i.e., that fraction not already blocked by deposits) determine the particle deposition rate in this bin (particles per m^2^ per s). Once deposited, particles grow, leaving bin 0 and entering bins with larger average radii, once their radius exceeds the value Δ*r*.

When discretizing, particles are evenly spaced within a bin, meaning *x*% of the bin width contains *x*% of the *N*_*i*_ particles in bin *i*. Fractional distance within a bin therefore equals the fraction of the particles in the bin that occupy that distance. If the particle radii grow by one-quarter of the thickness of a bin during a time step $\left(\frac{dr}{dt}\Delta t=0.25\Delta r_{\mathrm{bin}}\right)$ , then one-quarter of the particles in the bin should exit to the next bin, during that time step. The number of particles leaving a bin is therefore equal to the radial growth rate divided by the bin thickness, times the number density of particles in the bin. The differential equation for the number density of particles in bin *i* = 0 is therefore

2 $$\frac{dN_{0}}{dt}=f_{A}{\mathrm{q̇}}_{\mathrm{nuc}}^{\prime \prime }-\frac{\frac{dr}{dt}}{\Delta r_{\mathrm{bin}}}N_{0}$$

where the fraction of available surface area *f*_A_ = 1 – Σ_*i*_ *N*_*i*_π*r*_*i*_^2^. For bins with radii larger than $r_{\mathrm{nuc}}+\frac{\Delta r}{2}$ , there is no nucleation, only growth. Particles “enter” a bin by growing too large for the preceding smaller bin, and particles “exit” by growing too large and entering the next largest bin. Entry and exit rates follow the logic laid out in eq 2. For a bin *i* > 0, we therefore have

3 $$\frac{dN_{i}}{dt}=\frac{\frac{dr}{dt}}{\Delta r_{\mathrm{bin}}}\left(N_{i-1}-N_{i}\right)$$

The total number of bins and the bin thickness therefore determine the average radius of the largest bin, *i* = *n*_bins_.

4 $$r_{\max}=\Delta r\times n_{\mathrm{bins}}$$

No particles can exit this bin, leading to the following differential equation

5 $$\frac{dN_{N}}{dt}=\frac{\frac{dr}{dt}}{\Delta r_{\mathrm{bin}}}N_{n_{\mathrm{bins}}-1}$$

Consequently, the largest bin is not intended to contain an appreciable number of particles. If it does, this is a signal that more bins are needed for the simulation.

The simulation results for algorithm 1 are shown in Figure 2. The figure shows four PSD snapshots, each as a function of nondimensional time *t̂*, normalized by the total simulation time (4 s). The nucleation rate is constant and stops at $\mathrm{t̂}=\frac{1}{8}$ . The total number of particles remains constant for the remainder of the simulation. We normalized the *y*-axis data by dividing the number particles in each bin by the total number of particles deposited. Because the PSD shape is the point of emphasis, here, the actual particle concentrations are not of particular importance, and concentration differences between one algorithm and the next would only serve to distract from the salient features. For algorithm 1, even though the growth rate is the same for all particles, regardless of radius, the PSD in Figure 2 widens and flattens with time, indicating inconsistency with the underlying model assumptions. Particles prematurely exit the leading bin and linger in trailing bins. Moreover, while the constant growth rate $\frac{dr}{dt}=0.25\;\mu m\;s^{-1}$ implies a maximum radius of 1 μm after the 4 s-long simulation, we observe a significant population of particles with *r* > 1 μm and some small number of particles with *r* > 1.25 μm (see inset). Similarly, at the trailing edge, the last nuclei formed at $\mathrm{t̂}=\frac{1}{8}$ should have a radius of 0.875 μm at *t̂* = 1. Instead, Figure 2 shows an appreciable portion of the PSD with *r* ≤ 0.75 μm.

**Figure 2:**
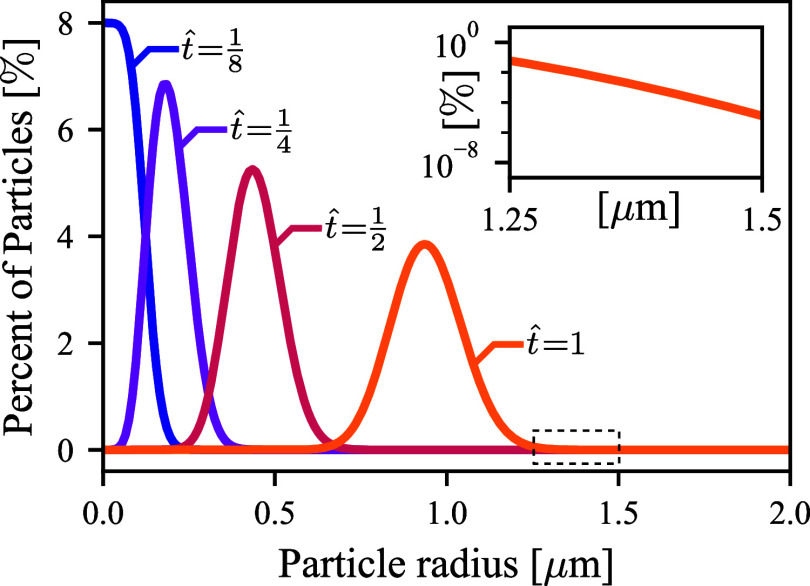
Simulated PSD snapshots using Algorithm 1: simple finite differencing. Nondimensional time *t̂* is scaled by the total simulation time (4 s). The observed flattening of the PSD shape with time is inconsistent with the model assumptions, and the maximum radius far exceeds the theoretical maximum of 1 μm (see inset), revealing shortcomings in this PSD tracking approach.

The errors of this algorithm are due to homogenization within the spatial mesh: for a bin *i*, the algorithm does not discriminate between particles that are barely larger than $r_{i}-\frac{\Delta r}{2}$ and those that are barely smaller than $r_{i}+\frac{\Delta r}{2}$ . In any bin, entering particles should be required to grow by Δ*r* before they can exit. Instead, homogenization in algorithm 1 dictates that as soon as *N*_*i*_ > 0 for a bin *i*, a fraction of the particles can immediately grow out of the bin and into bin *i* + 1. In the trailing bin, the only way all particles will leave a bin is if growth during a time step, $\frac{dr}{dt}\Delta t$ , exceeds Δ*r*. Otherwise, some fraction of the particles will always remain, reminiscent of Zeno’s paradox.

### 3.2 Algorithm 2: “Bookmarking” the PSD Upper and Lower Limits

To enforce maximum and minimum radii consistent with the model assumptions, algorithm 2 includes leading and trailing “bookmarks” that track the smallest and largest particle radii, respectively, as illustrated in Figure 3. The leading bookmark prevents particles from prematurely exiting the largest occupied bin and the trailing bookmark prevents particles from remaining too long in the trailing bin. Both bookmarks begin at *r*_0_ and advance at the rate $\frac{dr}{dt}$ . The leading bookmark moves immediately and the trailing bookmark moves once nucleation ceases (this last simplification is for easily interpretable results; other approaches can be implemented, if desired).

**Figure 3:**
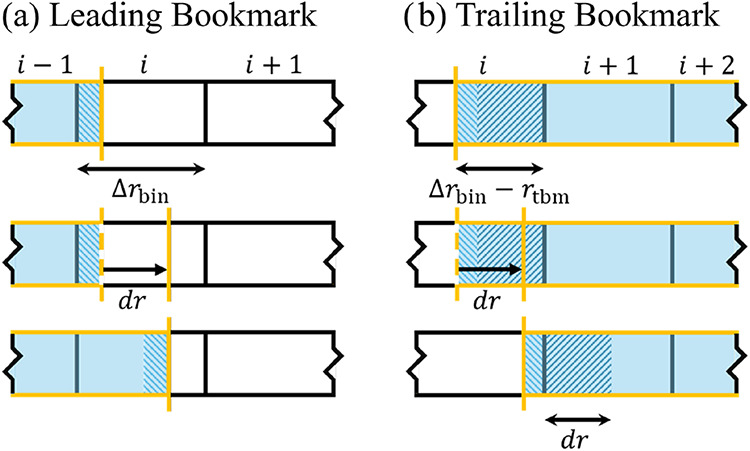
Bookmarks start and end in bin *i*. Each row depicts the same set of bins at different stages of a single time step. The top is the initial state, the middle is the change, and the bottom is the final state. The bookmark moves at the radial growth rate and its progress in single time step is *dr*. The patterned fills mark the portions of particles that begin the time step in the bin with the bookmark. (a) The leading bookmark prevents particles from prematurely exiting the bin. (b) The trailing bookmark controls the particle flux leaving the trailing bin. The fraction of particles that leave the trailing bin is equal to the darker patterned portion divided by the entire patterned portion.

Equations 2, 3, and 5 apply to bins with no bookmarks at time *t*. Modifications are required for any bin with a bookmark. The leading bookmark regulates the particle flux leaving the largest occupied bin, referred to as the leading bin. When the leading bookmark moves within a bin, no particles leave the bin. Particles enter from the previous bin as they did in eq 3

6 $$\frac{dN_{i,\mathrm{lbm}}}{dt}=\frac{\frac{dr}{dt}}{\Delta r_{\mathrm{bin}}}N_{i-1}$$

7 $$\frac{dN_{i,\mathrm{lbm}+1}}{dt}=0$$

where *i*,lbm is the leading bin index. Equations 6 and 7 prevent the “forward diffusion” of particles observed in Figure 2. The vertical yellow lines in Figure 3a mark the leading bookmark location.

The bookmark position reflects the fact that particles do not occupy all radii equally within the leading bin. Only the portion of the leading bin behind the bookmark is occupied, and particles will not leave a bin until the leading bookmark advances to the next bin. When the leading bookmark advances to the next bin, the new leading bin is initially unoccupied. The transition from zero flux to finite flux in this bin leads to large residuals and small time step when the bookmark crosses bin boundaries. The algorithm therefore approximates that a leading bookmark is always moving *within* a bin. The error introduced by neglecting the number or particles that move between bins during the same time step as the bookmark is negligibly small. The time step change as a bookmark approaches a bin boundary impacts the compuational time for this algorithm, as discussed below.

The trailing bookmark guarantees that particles do not reside in the trailing bin for longer than the prescribed residence time, $\frac{\Delta r}{\frac{dr}{dt}}$ . When the trailing bookmark moves within a bin, the fraction of particles that outgrow that bin equals the distance the radius grew in that time step divided by the distance between the right edge of the bin and the bookmark at the start of the time step

8 $$\frac{dN_{i}}{dt}=-\frac{\frac{dr}{dt}}{\Delta r_{\mathrm{bin}}-r_{\mathrm{tbm}}}N_{i}\left(t\right)$$

9 $$\frac{dN_{i+1}}{dt}=\frac{\frac{dr}{dt}}{\Delta r_{\mathrm{bin}}-r_{\mathrm{tbm}}}N_{i}\left(t\right)-\frac{\frac{dr}{dt}}{\Delta r_{\mathrm{bin}}}N_{i+1}\left(t\right)$$

where *r*_tbm_, as shown in Figure 3, is the relative distance between the trailing bookmark radius (as tracked in the solution vector) and the minimum radius of bin *i*. As with *r*_lbm_, the algorithm approximates that the trailing bookmark always moves *within* a bin.

The simulation in Figure 4a uses the bookmark approach and a constant nucleation rate. Results show that the bookmarks successfully enforce growth limits at the leading and trailing edges of the PSD. However, the algorithm fails when the nucleation rate varies with time, as shown in Figure 4b. Both simulations have constant growth rates and stopped nucleating at $\mathrm{t̂}=\frac{1}{8}$ . All profile widths in Figure 4 are invariant with time. However, the PSD profile changes with time in Figure 4b. As above, the flattening of the PSD within its bounds in Figure 4b stems from the assumption that particles are evenly distributed within bins.

**Figure 4:**
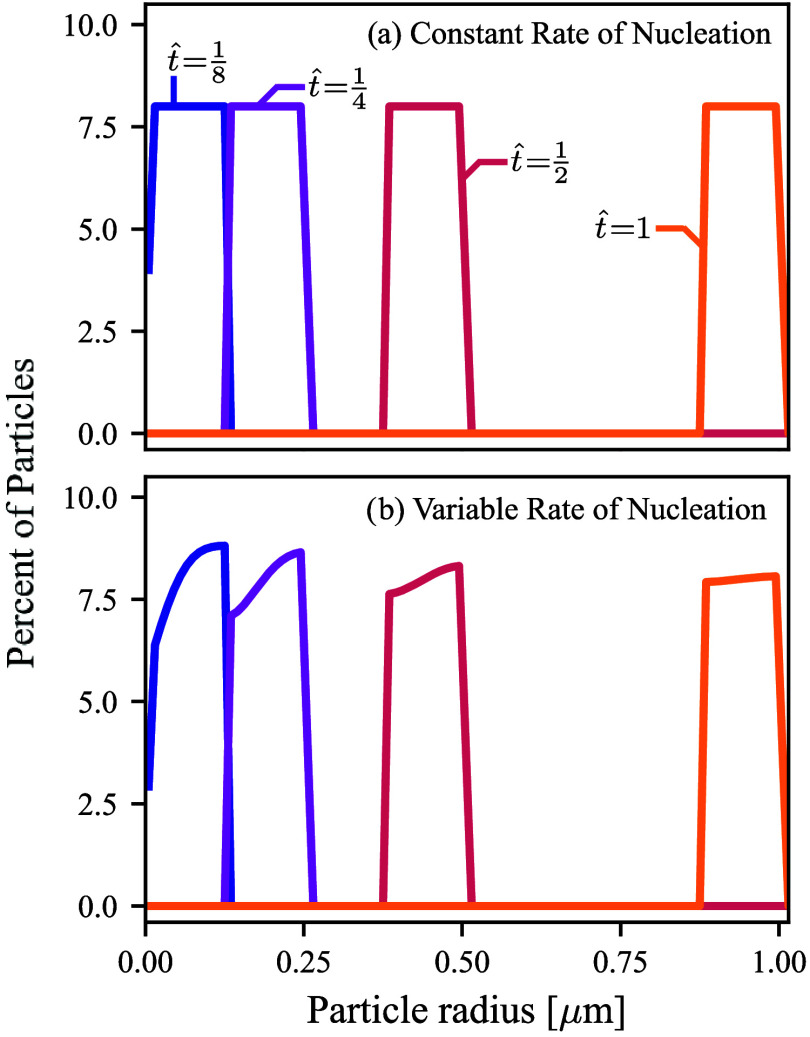
PSD snapshots during a simulation using algorithm 2: “Bookmarks.” (a) Constant nucleation rate. Bookmarks halt the premature flux of particles shown in Figure 2. (b) Variable nucleation rate. Here, the bookmarks enforce the correct minimum and maximum radius. However, the PSD flattening from Figure 2, while contained by the bookmarks, still occurs between these limits.

### 3.3 Algorithm 3: The “Moving Hopper” Model

In our model, nucleation is the sole mechanism capable of establishing or altering the PSD shape; growth shifts the entire profile while maintaining constant relative spacing between particle sizes. One way to visualize the deposition process is as a hopper above a conveyor belt. The hopper is positioned at the start of the belt and the nucleation rate determines how many particles flow through the hopper into the bins below. As the particles grow, the conveyor belt moves the bins and their particles to new sizes, while new bins appear under the hopper to receive nuclei. This is visualized in Figure 5a, where bins move down the conveyor belt at the growth rate. The particle radius in the bin directly under the hopper is *r*_nuc_, and each subsequent bin’s radius is calculated by adding Δ*r*_bin_ to the radius of the previous bin. Translating this concept directly into code, while accurate, would require the number of state variables to vary dynamically.

**Figure 5:**
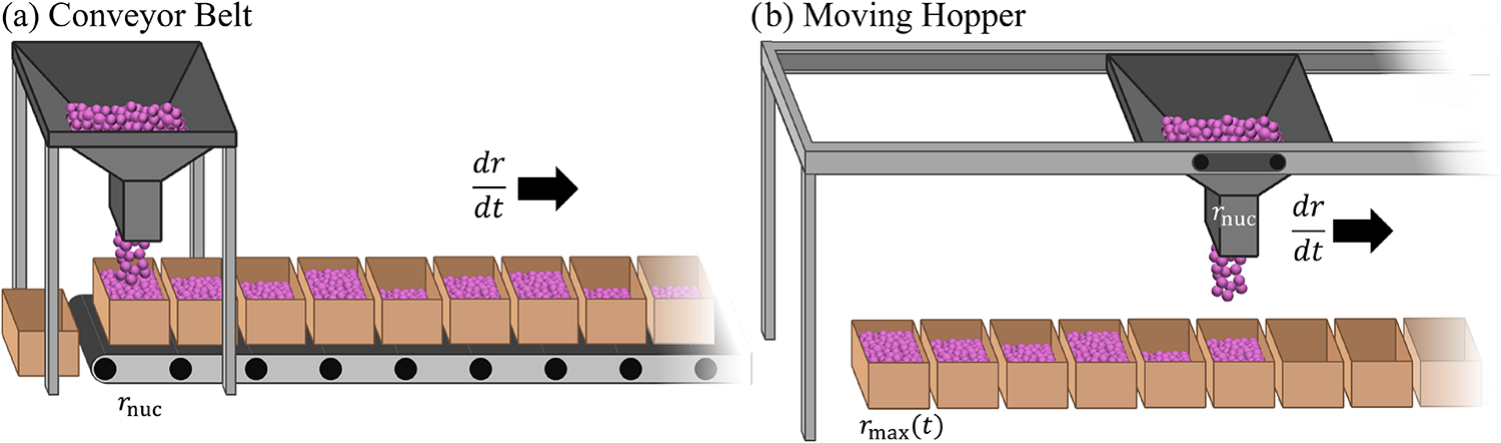
(a) A static hopper deposits particles while bins move on a semi-infinite conveyor belt below. All particles are deposited at the nucleation radius, and grow at the same rate, $\frac{dr}{dt}$ . The radius of each bin changes as the particles grow. (b) The simulation is re-envisioned, where hopper moves along semi-infinite tracks above unmoving boxes. The foundational concepts are the same as in (a), but this set up is easier to translate into code. The leftmost bin will always have the largest radius, and hopper always deposits particles at the nucleation radius.

To avoid dynamically resizing the solution vector, our approach re-envisions the coordinate system such that the deposited particles do not move between bins. Rather, *r*_*i*_, the average radius of particles in any bin *i* changes with time; *N*_*i*_, the number density of particles in bin *i* only changes when *r*_*i*_ = *r*_nuc_. Figure 5b illustrates the concept. The ‘hopper,’ indicating the bin where *r*_*i*_ = *r*_nuc_, begins at *i* = 0 at *t*_0_, and moves to the right (*i* > 0) at the growth rate $\frac{dr}{dt}$ . The number of particles in a bin can only change if the hopper is located over that bin at time *t* (note that the hopper can also remove particles due to stripping reactions; this is not implemented here, for simplicity). A local coordinate system moves with the hopper, defined by one additional variable: *r*_0_ the radius of bin zero at time *t*, which also corresponds to the maximum radius. Because the growth rate does not vary with particle radius in this simulation, all other radii between 0 ≤ *r*_*i*_ ≤ *r*_0_ are determined as

10 $$r_{i}=r_{0}-i\times \Delta r_{\mathrm{bin}}$$

Equation 10 is only needed during post processing, not during the simulation. The bin directly beneath the hopper at a given time is that with radius $0\leq r\leq r_{\mathrm{nuc}}+\frac{\Delta r_{\mathrm{bin}}}{2}$ , determined via modulus division. This leads to the following set of differential equations

11 $$\frac{dN_{i_{\mathrm{nuc}}}}{dt}=f_{A}{\mathrm{q̇}}_{\mathrm{nuc}}^{\prime \prime },\mathrm{For}\;i=i_{\mathrm{nuc}}$$

12 $$\frac{dN_{i}}{dt}=0,\mathrm{For}\;\mathrm{all}\;\mathrm{other}\;i$$

13 $$\frac{dr_{0}}{dt}={\mathrm{v̅}}_{\mathrm{dep}}{\mathrm{q̇}}_{\mathrm{growth}}^{\prime \prime }$$

where *v̅*_dep_ is the molar volume of the deposited material. Figure 6 shows the results of this algorithm. The shape of the PSD in Figure 6 is invariant, consistent with the model assumptions, even when *q̇*_nuc_^″^ varies with time. Moreover, the maximum radius at the simulation’s end equals the predicted size. Unlike in Figure 4b, there is no flattening of the PSD during the simulation, demonstrating a suitable approach for PSD tracking during HNG reactions.

**Figure 6:**
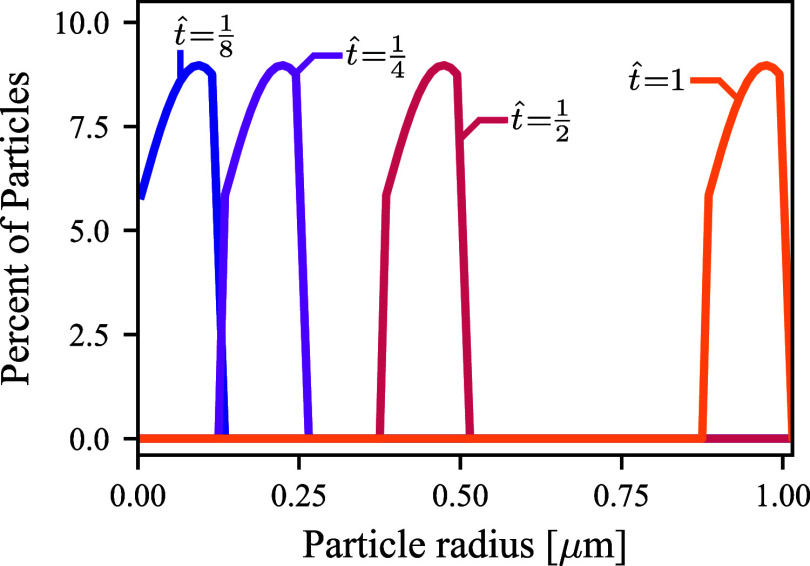
Four snapshots of the PSD during a simulation using algorithm 3 and a variable nucleation rate. The PSD is invariant, and moves at the radial growth rate.

### 3.4 Implications for Battery Simulations

The above results are intentionally technology agnostic so as to focus on the algorithmic components and the resulting inconsistency between model results and assumptions. However, it is worth exploring whether these inconsistencies meaningfully impact battery simulations. To test this we implemented the Li–O_2_ battery model in Lau and Archer,^15^ replicating the PSD shown in Figure 5b of that publication. The model assumes a constant intrinsic nucleation rate *q̇*_nuc_^″^ during the entire discharge (note that the nuclei deposition rate varies with the available surface area, *q̇*_nuc_ = *A*_avail_ *q̇*_nuc_^″^ and is time-varying). This provides a different set of conditions, with technologically relevant chemical rates, in which to evaluate the algorithm impact.

The resulting PSDs for our three algorithms above are shown in Figure 7, and the total discharge capacity, simulation time, and quantitative error calculations for each are provided in Table 1. The three algorithms give notably different PSD shapes. The “bookmark” approach from algorithm 2 fared the worst, failing to match the overall discharge capacity and taking the longest time to run due to residuals swelling as bookmarks approach bin boundaries. This issue was exacerbated by the need for two pairs of bookmarks, one for each section of nucleated particles. Algorithm 3, the “moving hopper” model correctly predicts the PSD shape and discharge capacity from the reference publication,^15^ and takes only 146 ms longer to run than algorithm 1. This is a significant increase (76%) relative to the algorithm 1 simulation time of 192 ms. However, as additional model equations are added to track electrolyte chemistry, electric potential, and other variables, the additional governing equation of algorithm 3 will have a lower relative impact.

**Table 1:** Performance Comparisons for the Three Algorithms in Replicating the Results from Lau and Archer^15^ ^,^ ^a^

algorithm	capacity [mAh]	run time [s]	avg. error [%]
1	2.536	0.192	66.49
2	2.478	0.961	66.51
3	2.619	0.338	–

a Algorithm 3 is the baseline for the percent error calculation, shown in eq 14. The simulated discharge capacity in Lau and Archer was roughly 2.6 mAh.

**Figure 7:**
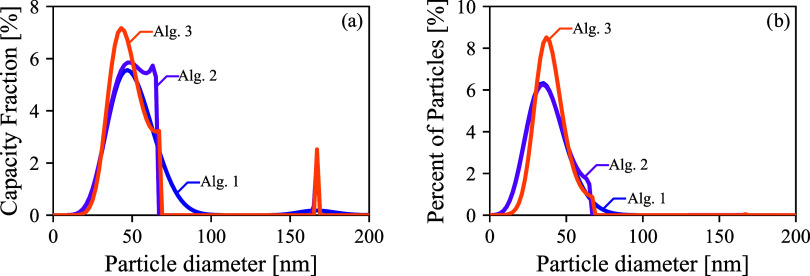
Final PSD snapshots after reproducing the simulated discharge described in Archer and Lau.^15^ Results presented as a fraction of (a) total capacity and (b) total number of particles. The particles deposited at the onset of discharge are negligible as a percentage of total particles, but represent a significant capacity fraction because of their large diameter. Algorithm 1 allows particles to diffuse into a normal distribution, including the cluster nucleated at the onset of discharge. Algorithm 2 uses “bookmarks” to constrain peak broadening, but allows flattening within the peak. Algorithm 3 employs the "moving hopper" model and matches the distribution from Archer and Lau. Both algorithms 2 and 3 preserve the shape of the initial cluster.

The average error in Table 1 represents the average percent mismatch at radius *r*_*i*_, relative to algorithm 3, using the a sum of squared residuals

14 $$\mathrm{Er}r_{j}=\frac{1}{n_{\mathrm{bins}}}\sum_{i=0}^{n_{\mathrm{bins}}}w_{i,j}{\left(\frac{N_{i,j}-N_{i,3}}{N_{i,3}}\right)}^{2}$$

where *N*_*i*,*j*_ is the number density (particles per unit area) of deposited particles having radis *r*_*i*_ using algorithm *j*, and where each error is weighted by *w*_*i*,*j*_, the average fraction of particles with radius *r*_*i*_

15 $$w_{i,j}=\frac{1}{2}\left(\frac{N_{i,j}}{\sum N_{i,j}}+\frac{N_{i,3}}{\sum N_{i,3}}\right)$$

For radii where *N*_*i*,3_ = 0, but *N*_*i*,*j*_ ≠ 0 for algorithm 1 or 2, the local error contribution (before weighting) is set to 100% to avoid division by zero. In Table 1, we observe roughly the same average errors for algorithms 1 and 2. This reflects the fact that the “bookmarking” in algorithm 2 does not prevent so-called “diffusion” between radial bins; it only constrains it, so that particles pile up at the leading bookmarks. Moreover, the average error is proportional to the area between the algorithm’s PSD and that of algorithm 3. Visual inspection of Figure 7a shows roughly equal areas for algorithms 1 and 2 on either side of the primary maximum radius from algorithm 3, consistent with the error results in Table 1.

Although algorithms 1 and 2 nearly match the overall capacity from the reference publication, the differences in PSD will have increasing impact as simulations are used to predict extended cycling in realistic situations. For example, if the battery is not fully recharged (i.e., deposits not fully removed) before charging resumes, the particles at the low-diameter end of the PSD will erode, but the high-diameter portion of the PSD would remain. The shape of the PSD when discharge resumes will impact the available surface area for growth, the resulting degree of supersaturation, and the particle sizes that develop during discharge, impacting subsequent battery performance.

## 4 Conclusions

As heterogeneous nucleation and growth phenomena become increasingly important in a range of energy storage and conversion technologies, in batteries and beyond, attention to the particle size distribution tracking will require greater attention. In this study, initial attempts at tracking particle growth using standard spatial discretization methods resulted in flattening between bins that distorted the PSD. To solve this type of anomalous “particle diffusion” between discretized radial bins, we propose here a “moving hopper” algorithm that controls which bin particles are deposited into instead of moving particles between bins. Once deposited, a particle remains in a given discretized bin/position within the solution vector. Rather than moving particles to different bins as they grow, this approach alters the average radius associated with each bin. The approach only requires two differential equations, at any given time–one rate for the bin where deposition occurs and one for the growth rate of the largest particle–supporting computational efficiency.

The model implemented here assumes that the particles deposit and grow as hemispheres with a uniform radial growth rate $\frac{dr}{dt}$ for all particles, regardless of size. However, the implications of our findings are relevant for any particle shape and even if the radial growth rate is a function of radius. Any finite differencing scheme that moves particles between discrete bins and assumes even particle distribution within bins will cause the PSD to flatten over time. The algorithm here also provides a pathway for situations where *q̇*_growth_^″^ does vary with particle radius. In this situation, particles in bin *i* are related not just by size, but also temporally: particles deposited at the same time will evolve in a uniform manner. In this situation, tracking *N*_*i*_ and *r*_*i*_ for every bin will permit radially dependent growth rates, but with added computational cost.

While implementing additional governing equations can add complication (particularly if integrating electrochemical models into multiphysics software routines) and computational cost, for the algorithms suggested here we find minimal cost. Our proposed algorithm adds a single additional variable/governing equation (eq 13) that depends only on the chemical growth rate *q̇*_growth_ and can be readily implemented as a user-defined function in multiphysics software. Our calculations show only a 146 ms increase in computational time, relative to the standard spatial discretization approach. Moving forward, we encourage research to ensure that adopted PSD tracking approaches appropriately preserve the underlying model assumptions, and to clearly document these algorithms in relevant publications.

To support open science practices, the code for the three algorithms is publicly available on GitHub at https://github.com/coresresearch/2026_Particle-Tracking.git.
